# Abnormal coexistence of unipolar, bipolar, and threshold resistive switching in an Al/NiO/ITO structure

**DOI:** 10.1186/1556-276X-9-268

**Published:** 2014-05-29

**Authors:** Xin-Cai Yuan, Jin-Long Tang, Hui-Zhong Zeng, Xian-Hua Wei

**Affiliations:** 1 State Key Laboratory Cultivation Base for Nonmetal Composites and Functional Materials, Southwest University of Science and Technology, Mianyang 621010, People's Republic of China; 2 School of Science, Southwest University of Science and Technology, Mianyang 621010, People's Republic of China; 3 State Key Laboratory of Electronic Thin Films and Integrated Devices, University of Electronics Science and Technology of China, Chengdu 610054, People's Republic of China

**Keywords:** Resistive switching, Thin film, Interface, Indium tin oxide substrate

## Abstract

This paper reports an abnormal coexistence of different resistive switching behaviors including unipolar (URS), bipolar (BRS), and threshold switching (TRS) in an Al/NiO/indium tin oxide (ITO) structure fabricated by chemical solution deposition. The switching behaviors have been strongly dependent on compliance current (CC) and switching processes. It shows reproducible URS and BRS after electroforming with low and high CC of 1 and 3 mA, respectively, which is contrary to previous reports. Furthermore, in the case of high-forming CC, TRS is observed after several switching cycles with a low-switching CC. Analysis of current-voltage relationship demonstrates that Poole-Frenkel conduction controlled by localized traps should be responsible for the resistance switching. The unique behaviors can be dominated by Joule heating filament mechanism in the dual-oxygen reservoir structure composed of Al/NiO interfacial layer and ITO. The tunable switching properties can render it flexible for device applications.

## Background

Binary transition metal oxides like NiO, TiO_2_, and ZnO have attracted much attention in the field of resistive switching due to simple constituents, low deposition temperature, and compatibility with complementary metal-oxide semiconductor technology [1,2]. Interestingly, different resistive switching behaviors have been found in metal/NiO/metal when different electrode materials were employed, such as Pt, Ag, Cu, and Al [3-6]. Lee et al. have found unipolar resistive switching (URS) in Ag(Cu)/NiO/Pt due to the formation of an oxide layer at the metal/NiO interface [3]. Chiang et al. have demonstrated that bipolar resistive switching (BRS) in Al/NiO/indium tin oxide (ITO) as Al/NiO interfacial reaction region combined with ITO can form a dual-oxygen reservoir structure [4]. In addition, Ni/NiO/Ni with different device structure exhibits URS and BRS modes, separately driven by electrochemical- and thermal-based mechanisms [7]. Threshold resistive switching (TRS) and URS in NiO thin film were also found at different measuring temperatures by Chang et al*.*[8]. The occurrence of TRS and BRS in Mn-doped ZnO device was found with a higher CC by Yang et al. due to Joule heating [9]. More recently, the transition from URS to TRS can be tuned by the strong electron correlation through controlling the film stoichiometric ratio [10]. Moreover, the coexistence of different resistive switching behaviors has been found in many materials such as BiFeO_3_[11,12], HfO_2_[13,14], SrTiO_3_[15], ZnO [16-18], diamond-like carbon [19], and TiO_2_[20]. The choice of switching modes can broaden device applications and enable large flexibility in terms of memory architecture [15]. Generally, URS was preferred under high compliance current (CC), while BRS under low CC. In this letter, we present an abnormal coexistence of URS with a low CC and BRS under high CC in the same Al/NiO/ITO device. Meanwhile, TRS was also observed by reducing the switching CC to forming CC. The Joule heating filament mechanism in a dual-oxygen reservoir structure composed of Al/NiO layer, and the ITO substrate was responsible for the abnormal resistance switching.

## Methods

**Figure 1:**
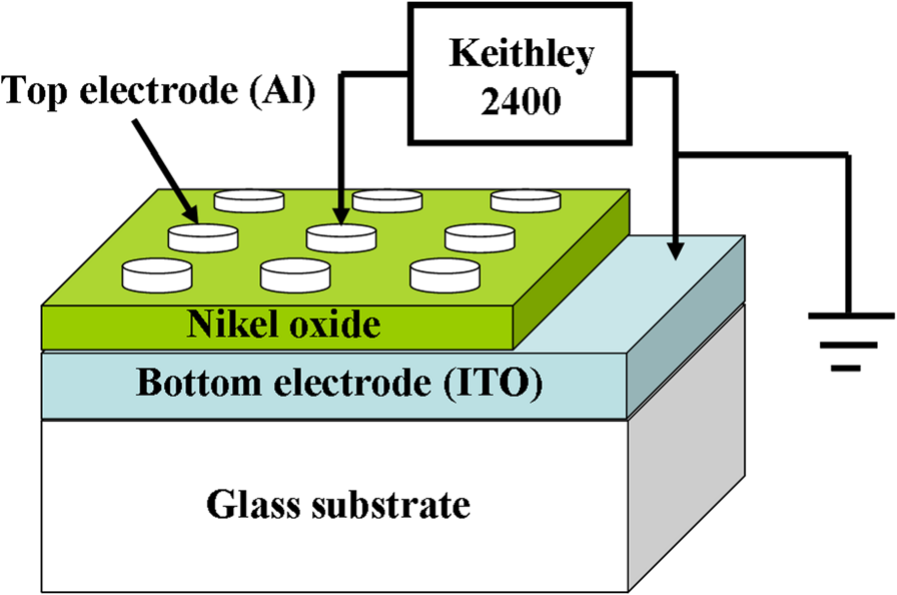
Schematic of the Al/NiO/ITO device and setup for measurement.

NiO thin films were fabricated on ITO substrates by sol-gel process [21]. Nickel acetate tetrahydrate was used as a metal source, and 2-methoxyethanol and ethanolamine as solvent and stabilizing agent, respectively. Then, the mixed solution was stirred for an hour at 80°C to obtain a homogeneous stacked solution. The precursor solution (0.18 ml^−1^) was drop-casted on cleaned ITO substrate and rotated at 3,000 rpm for 30 s using a spin coater. After spin coating, the sample was dried on a hot plate at 120°C for 5 min to evaporate the solvent and remove organic residuals. Thin films were synthesized by repeating the above processes followed by annealing in air ambient at 475°C for 2 h. Crystal structures were determined by X-ray diffraction (XRD; Philips X'pert MPD Pro, Amsterdam, Netherlands) with Cu K_α_ radiation (*λ* = 0.15406 nm), and atomic force microscopy (AFM; Seiko SPI 3800, Chiba, Japan) was used to evaluate the surface morphology. Circular top electrodes of Al and Au with diameter of 500 μm were deposited by vacuum thermal evaporation through a shadow mask. A schematic of the Al/NiO/ITO device is shown in Figure 1. The transport properties of the device were characterized using a Keithley 2400 SourceMeter (Cleveland, OH, USA) at room temperature with a sweeping voltage applied to the Al top electrode while the ITO bottom electrode was grounded. To prevent disturbances from light and electromagnetic waves, current-voltage (*I*-*V*) measurements were performed in a metal dark box.

## Results and discussions

**Figure 2:**
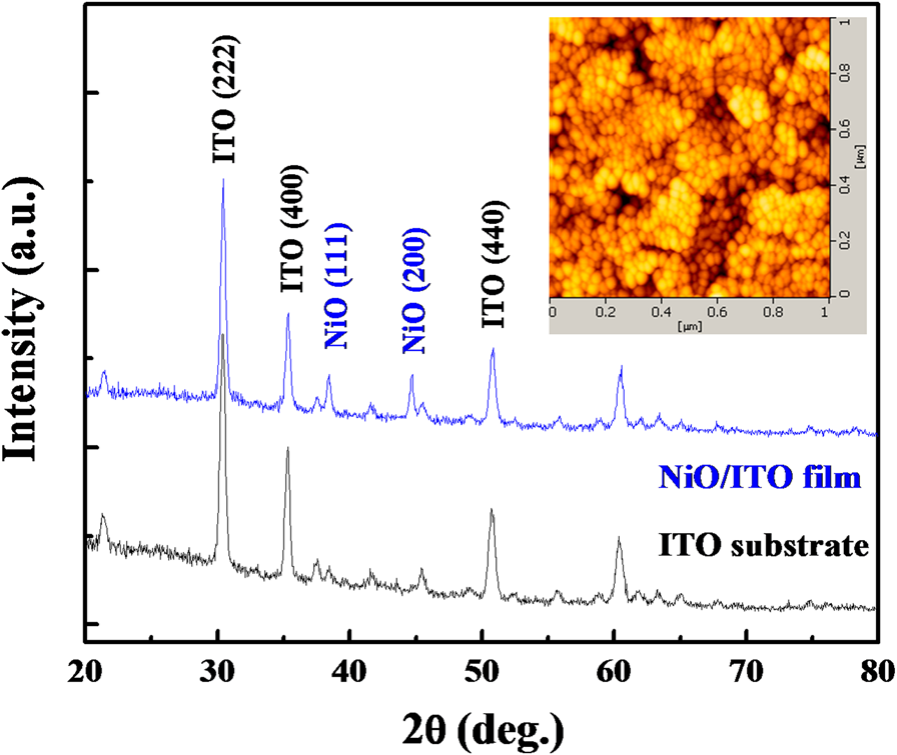
**XRD pattern of NiO film on ITO substrate annealed at 475°C in air ambient.** Inset shows the AFM test of the NiO film.

Figure 2 compares the XRD pattern of the NiO/ITO film and the ITO substrate. In addition to those diffraction peaks from the ITO substrate, only NiO (111) and NiO (200) peaks were observed, suggesting that the NiO film has been successfully fabricated. The inset demonstrates the AFM image of the NiO thin films, in which the surface roughness of the films has a root-mean-square value of 3 nm, and the average grain size is about 30 nm, indicating that the film had a smooth surface relatively.

**Figure 3:**
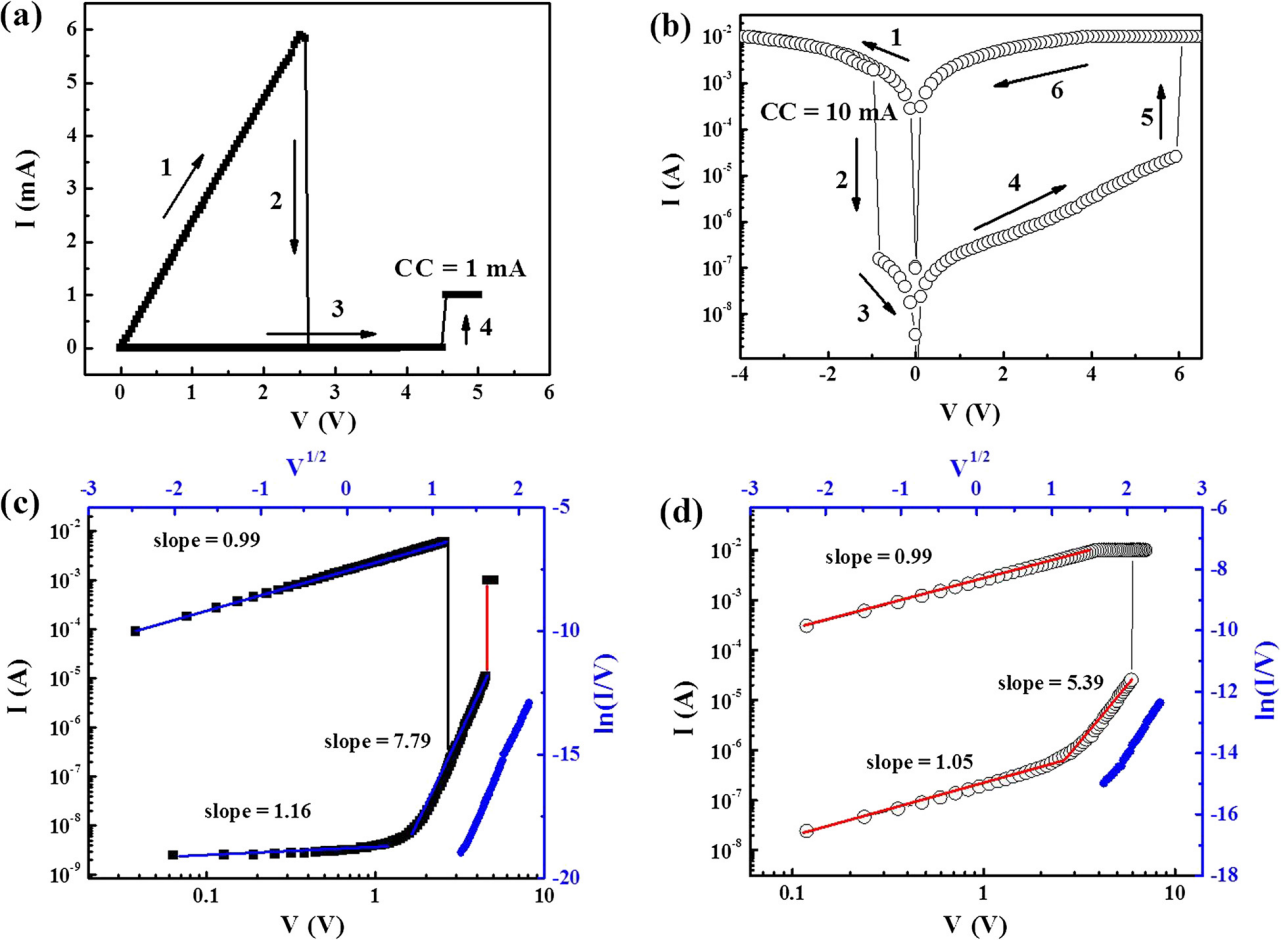
***I*-*V *characteristics test of URS and BRS in the Al/NiO/ITO.** The forming process is showed at the inset. **(a)** URS *I*-*V* curve. **(b)** BRS log(*I*)-*V* curve . **(c)** URS log(*I*)-log(*V*) curve; the blue axis shows the fitting log(*I*/*V*) − *V*^1/2^. **(d)** BRS log(*I*)-log(*V*) curve; the blue axis is the log(*I*/*V*) − *V*^1/2^.

Figure 3a,b shows two linear URS and BRS of *I*-*V* curves in the same Al/NiO/ITO device. The insets demonstrate electroforming processes with CC of 1 and 3 mA, respectively, which occurred respectively at about 4.9 and 7.8 V with an abrupt current increase up to CC. After the forming process, the device was transformed from initial high resistance state (HRS) to low resistance state (LRS), and conductive filaments were formed. For URS, a reset process (LRS to HRS) was shown at the voltage of about 2.6 V. By applying a higher positive bias at about 4.5 V, the set process (HRS to LRS) was found to increase the current up to the CC (1 mA). For BRS, the voltage bias was swept in a sequence of 0 → negative → positive → 0 as indicated by the arrows in Figure 3b. The negative bias was defined as the current flowing from the ITO bottom electrode to the Al top electrode. *I*-*V* hysteresis was pronounced when the CC during switching process (10 mA) was larger than the forming CC. The set and reset voltages were about 6.0 and −1.0 V, respectively, and the ON/OFF ratio at 0.12 V was larger than 10^4^, which was close to that of URS in the same device.

In order to get further understanding of the mechanism of the URS and BRS behaviors, two linear fitting curves of log(*I*)-log(*V*) were separately depicted in Figure 3c,d. At LRS for both URS and BRS, the linearity curves with slopes of almost 1 indicate ohmic conduction behavior, which were typically due to the formation of conductive filaments in NiO. However, at the HRS state, the *I*-*V* characteristic was more complicated and could be divided into two parts. At low voltages, the *I*-*V* curve was linear, corresponding to the ohmic mechanism region. At high voltages, the slope was much larger than 1, indicating that the conduction mechanism was dominated by trap-limited space charge-limited current (SCLC) conduction. In addition, by fitting ln(*I*/*V*) ~ *I*^1/2^ curve in HRS as shown in the blue lines, it seems to be governed by Poole-Frenkel (PF) emission that involves thermal effect and trap sites such as oxygen vacancies. The detailed mechanism of resistive switching based on these effects will be explained later.

**Figure 4:**
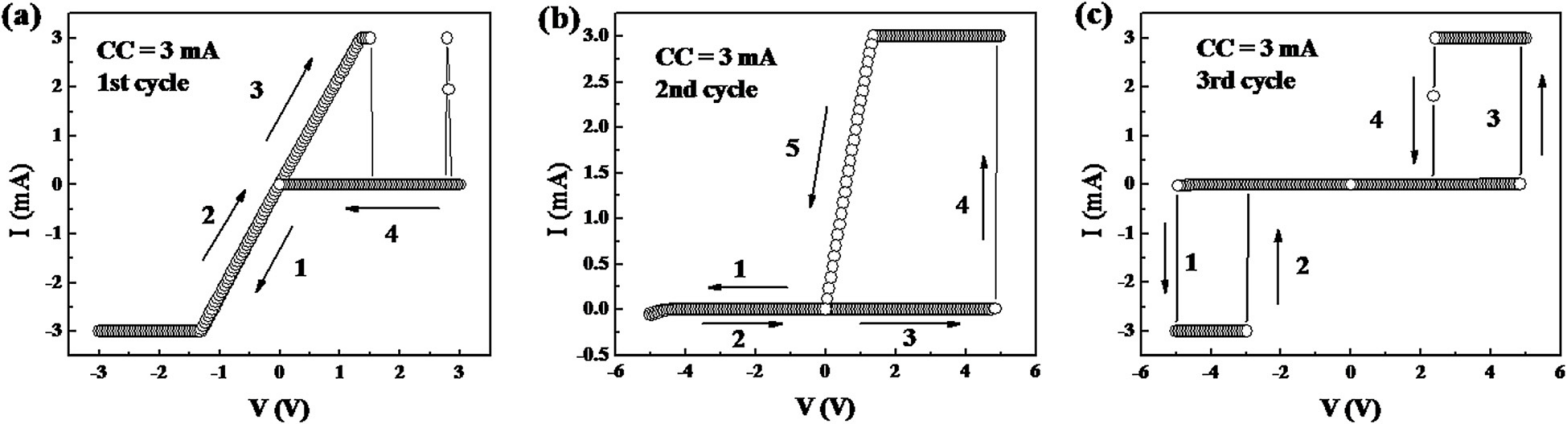
**Resistive switching evolution with the same CC (3 mA) of forming and switching. (a)** The first *I*-*V* cycle. **(b)** The second *I*-*V* cycle. **(c)** The third *I*-*V* cycle.

When the CC during switching process of the *I*-*V* hysteresis measurement was reduced to forming CC, the switching behaviors show a series of transition after several cycles. Figure 4a exhibits the *I*-*V* curve for the first switching cycle with two CCs of 3 mA. At negative bias, the device was always in LRS. At positive bias, it was an URS-like switching that the device was converted to HRS at about 1.5 V. For the second cycle as shown in Figure 4b, it shows a BRS-like characteristic, except that there is no obvious set process. The ON/OFF ratio at the negative bias was very small since the device was almost kept at HRS regardless of swept direction. It was quite intriguing that a typical TRS was reproducible from the third cycle as shown in Figure 4c. The device switched from HRS to LRS with abrupt increase of current which occurred at −5.0 V and returned back to HRS at −3.0 V. The same behaviors were observed at positive threshold voltages of 4.9 and 2.3 V.

**Figure 5:**
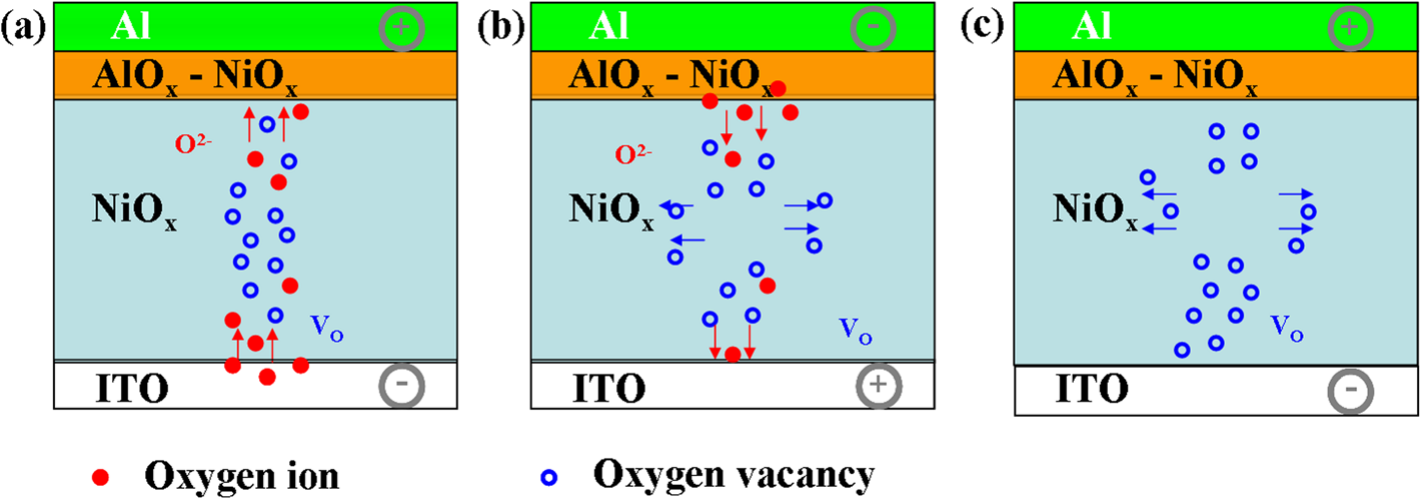
**Oxygen migration at the top and bottom interfaces of the NiO layer and Joule heating effect. (a)** BRS set process. **(b)** BRS reset process. **(c)** URS reset process.

From the viewpoint of driving force, URS is dominated by Joule heating with a high CC and BRS by electrical field with a low CC [15,16,19,20,22]. A higher CC means a higher current that generated more Joule heating, which could be responsible for the mechanism of rupturing the conductive path in the URS. In general, BRS in oxide memory devices was attributed to the drift of oxygen ions. The abnormal results in this work might be ascribed to the device structure of NiO sandwiched between dual-oxygen layers, as shown in Figure 5. Chiang et al. have identified Al_2_O_3_ oxide layer at the interface between an Al electrode and NiO by X-ray photoelectron spectroscopy (XPS) [4]. It is easily understood in terms of standard enthalpy change of formation of oxides (NiO:ΔH_f_^298^ ~ −244.3, Al_2_O_3_:ΔH_f_^298^ ~ −1,669.8) [3,23,24]. Here, we need to point out that the resistive switching behavior was not found in the Au/NiO/ITO structure (not shown here), suggesting that the Al/NiO interface should play a decisive role in resistive switching. The formation of interfacial oxide layer can act as an oxygen reservoir, in which oxygen ions will migrate under applied electric field. In this case, the switching was decided by the exchange of oxygen ions at the interface between the interfacial layer and NiO [4,25]. The exchange leads to the construction/rupture of the conducting paths composed of oxygen vacancies. Similarly, it was found by time-of-light secondary ion mass spectroscopy that ITO can also be considered as another oxygen reservoir [10]. Therefore, a dual-oxygen reservoir structure model should be proposed since any of the Al/NiO interfacial oxide and ITO can provide a chance to exchange oxygen ions to construct a conduction channel. For the set process of BRS, the conductive filaments were formed, owing to the migration of the oxygen ions from the ITO bottom electrode to the Al/NiO region as shown in Figure 5a. At opposite bias, the possibility of reset process would be small due to the migration of oxygen ions from the Al/NiO interface to ITO to form the conductive filament as shown in process 1 (0 to −4 V) in Figure 3b. However, the occurrence of the reset process of BRS at −4 to 0 V is different from that of the typical BRS behavior in single oxide layer. In the dual-oxygen reservoir structure model, no matter what the direction of the electric field is, there is always oxygen provision to form the filaments. Therefore, the unusual reset process demonstrates that Joule heating rather than electric field effect might be the main factor in rupturing the conductive filaments as shown in Figure 5b. It is also the reason that BRS is preferred with higher CC to generate enough Joule heating to overcome the effect of electric field on oxygen ion movement. Similarly, the set process of URS is mainly dominated by the oxygen migration from ITO to Al/NiO interface. Nevertheless, a low CC can trigger the occurrence of reset process during the measurement of URS because no additional electromigration happens as shown in Figure 5c. If switching CC is reduced to 3 mA, it means there is insufficient heating to rupture the same thick or dense filaments at the same forming process as the BRS behavior. This would lead to unstable resistive switching as shown in Figure 4a,b. At last, it will evolve to a volatile TRS due to a spontaneous rupture of filaments of insufficient heat dissipation induced by the Joule heating [8].

## Conclusions

NiO thin films were prepared by solution route with nickel acetate as the metal source. By control forming and switching CC, URS, BRS, and TRS were found in the same Al/NiO/ITO device. URS existed at low-forming CC, while BRS at high-forming CC, which was different from previous reports. From the fitting curves of *I*-*V*, the HRS at low voltage and LRS were dominated by Ohmic conduction, and the HRS at high voltage could be attributed to the PF emission that involves thermal effects and trap sites such as oxygen vacancies. The switching mechanism was discussed based on the dual-oxygen reservoir structure model in which the ITO electrode and Al/NiO interface acts as the oxygen reservoirs. No matter what the direction of the electric field is, the dual-oxygen reservoir structure will support the oxygen vacancies to form the conductive filaments. The reset process indicates that Joule heating might be the main factor in rupturing the conductive filaments. When the forming and switching CC was equal, we found TRS after several loop tests. It was caused by spontaneous rupture of the filaments of insufficient heat dissipation at higher CC due to the Joule heating. The tunable switching properties would enable large flexibility in terms of device application.

## Competing interests

The authors declare that they have no competing interests.

## Authors' contributions

XCY and XHW carried out the sample preparation, participated on its analysis, performed all the analyses, and wrote the paper. JLT and HZZ provided useful suggestions and helped analyze the characterization results. All authors read and approved the final manuscript.
